# Health Status Is Affected, and Phase I/II Biotransformation Activity Altered in Young Women Using Oral Contraceptives Containing Drospirenone/Ethinyl Estradiol

**DOI:** 10.3390/ijerph182010607

**Published:** 2021-10-10

**Authors:** Gerda Venter, Carien L. van der Berg, Francois H. van der Westhuizen, Elardus Erasmus

**Affiliations:** Human Metabolomics, Faculty of Natural and Agricultural Sciences, North-West University (Potchefstroom Campus), 11 Hoffman Street, Potchefstroom 2531, South Africa; carien.vanderberg@nwu.ac.za (C.L.v.d.B.); francois.vanderwesthuizen@nwu.ac.za (F.H.v.d.W.)

**Keywords:** biotransformation, oral contraceptives, fatigue, health status, oxidative stress

## Abstract

Combined oral contraceptive (COC) use has been associated with various adverse effects. Formulations containing drospirenone (DRSP) and ethinyl estradiol (EE) are generally regarded as milder COCs. Whether long term use of these pills indeed has a low health risk remains questionable. COC use may affect the biotransformation balance by increasing the toxic load or by interfering with the pharmacokinetics of other drugs. This may negatively impact overall health via the production of toxic biotransformation metabolites and induction of oxidative stress. Although individual enzymes involved in biotransformation are known to be regulated by COCs, the effect of COC use on the overall liver biotransformation efficiency has not been reported. Here, we evaluated the general subjective health status and overall liver biotransformation efficiency of healthy young women who were either long term chronic users of COCs containing DRSP/EE, or who were not using any hormonal products. COC users suffered from moderate to severe fatigue and reported more health-related symptoms. Furthermore, phase I (CYP1A2) activity was reduced whereas phase II conjugation reactions (glucuronide conjugation and glycine conjugation) were increased in COC users. Finally, serum peroxide levels were markedly elevated and antioxidant capacity of plasma was reduced in COC users. COCs containing DRSP/EE may, therefore, adversely affect health status and disturb the balance between phase I and II biotransformation reactions. These effects may be mediated by oxidative stress.

## 1. Introduction

Oral contraceptive pills account for 16% of all contraceptive methods used, thereby being the fourth most used method of contraception worldwide. In Southern Africa the pill is used third most, whereas in Europe and North America the pill appears to be the most used method of contraception, and globally its use seems to be increasing [1]. Combined oral contraceptives (COCs) consist of a progestin combined with a synthetic estrogen, such as ethinyl estradiol (EE). Although the primary application of COCs is for the prevention of pregnancy, these products are also being prescribed to treat various conditions including irregular periods, acne, hirsutism, and endometriosis and dysmenorrhea (menstrual cramps) [2,3,4]. Therefore, because women use COCs not only for contraception, the number of women using COCs may be even higher than what is currently estimated.

Although COCs are relatively commonly used for either contraceptive or non-contraceptive purposes, their use has been associated with a number of adverse effects. These include increased blood pressure, weight gain, fluid retention, reduced bone density, and increased risk for vascular disease and thrombosis [5,6,7]. Other, less severe side effects include headache, nausea, pelvic pain, and breast tenderness [8,9,10]. A more serious risk factor associated with COC use is the development of breast and cervical cancer [11], although this fact seems to be often ignored by users and physicians. In fact, high COC user satisfaction with regard to menstrual cycle-related symptoms seem to overshadow the potential health risks associated with COC use [12].

COC formulations have been adapted in recent years in order to reduce the number or severity of side effects and increase the therapeutic benefits. Fourth generation formulations containing drospirenone (DRSP) and EE are generally regarded as milder COCs [13,14] because they have been shown to improve symptoms associated with endometriosis and dysmenorrhea, premenstrual syndrome (PMS), acne, and hirsutism, with no increase in body weight, fluid retention, or blood pressure [4,15,16,17,18,19]. As a result, a large proportion of young COC users in South Africa, and worldwide [20], uses products containing DRSP/EE. Whether long term use of these pills indeed have a low health risk remains questionable, however. Women treated with DRSP/EE have been shown to have increased levels of thrombin, fibrin turnover F1 + 2, and D-dimer, in addition to reduced anticoagulatory factors [20], thereby increasing their risk to develop venous thromboembolism (VTE). In addition, DRSP/EE was also linked to elevated lipid-peroxidation which can consequently increase cardiovascular risk [21]. Although some studies indicate that the risk for VTE with long-term use of COCs containing DRSP/EE is comparable with that of other COCs [22,23,24], it is still increased compared to COC non-users [25]. Furthermore, headache, migraine, nausea, and depression are still listed as common side effects experienced by users [26]. Pathophysiological mechanisms have been described for many of the adverse effects associated with COC use; however, some are still not entirely understood. Previous studies have clearly indicated that COC use is associated with oxidative stress [27], although neither the origin nor the clinical implication of this has been investigated further.

After oral ingestion, the synthetic hormones contained in COCs are absorbed and undergo extensive first pass metabolism in the gut and liver, and only a fraction of the original dose is directly bioavailable [28]. Subsequent metabolism of these hormones involves both phase I and phase II biotransformation reactions. Phase I reactions involve oxidation, reduction, and hydrolysis of the xenobiotic and may yield intermediates that are more reactive than their parent molecules, in addition to reactive oxygen species (ROS). Phase II reactions include the conjugation reactions during which the reactive intermediates from phase I are converted into water-soluble products that can be excreted in the urine and bile. Efficient biotransformation is critical for overall health, and imbalances between phase I and phase II biotransformation have been linked to various diseases [29]. COC use may affect this balance by increasing the toxic load or by interfering with the pharmacokinetics of other drugs [28], and negatively impact overall health via the production of toxic biotransformation metabolites and induction of oxidative stress. Although the synthetic hormones in COCs have been shown to alter the expression of individual biotransformation genes, reports on the effect of COC use on the overall liver biotransformation efficiency are lacking.

The current study, therefore, aimed to determine the impact of the long-term chronic use of COCs containing DRSP/EE on the general subjective health status and the overall liver biotransformation efficiency of healthy women between 18 and 35 years of age. We found that women using the COC reported a higher level of fatigue and complained more frequently of health-related issues. In addition, phase I biotransformation was inhibited, whereas phase II activity was increased. These effects were accompanied by oxidative stress.

## 2. Material and Methods

### 2.1. Recruitment of Participants and Sample Collection

Ethical approval was obtained from the Health Research Ethics Committee (HREC) of the North-West University, South Africa (NWU-00344-16-A1). Participants between 18 and 35 years were recruited between April 2017 and October 2019 from the general public either by placing advertisements/flyers at a number of pharmacies, doctor’s rooms, supermarkets, and shopping centers in the Potchefstroom urban areas, or by placing an advertisement on social media platforms of the North-West University (NWU). Volunteers were asked to complete an eligibility questionnaire containing all the inclusion and exclusion criteria in the form of a checklist. Exclusion criteria were body mass index (BMI) ≥ 30, human immune deficiency virus (HIV) positive, irregular menstrual cycle, pregnancy or breastfeeding in the past six months, COC/hormone use in the past four years (controls), use of COC formulations other than DRSP/EE, using the COC for less than 3 months, self-reported indications of diabetes, liver or kidney disease, asthma, other chronic diseases, chronic medication use, and smoking more than 5 cigarettes/day. Power calculations indicated that a minimum sample size of 20 was required. In total, 49 eligible Caucasian women were included in the study (25 controls, 24 COC users). The COC used contained either 3 mg DRSP and 0.03 mg EE (*n* = 8), or 3 mg DRSP and 0.02 mg EE (*n* = 16).

Eligible volunteers were invited to an information session and written informed consent was obtained after at least one week consideration time. Participants were provided with a biotransformation and oxidative stress status (BOSS) test kit, medical symptoms questionnaire, lifestyle questionnaire, food frequency questionnaire, and revised Piper fatigue scale, along with proper instructions to use the kits (sampling) and to complete the questionnaires. All participants underwent HIV testing at the Student Counselling and Development division of the NWU Health Care Centre. Participants were requested to contact the research team on the first day of the menstrual cycle in which they were planning to perform the test. Loading tests (for evaluation of biotransformation efficiency) and sample collection were undertaken as described in Erasmus et al. [30] in the luteal phase of the menstrual cycle. Because cycle length varies between women, individual test dates were calculated as average cycle length minus eight days. This means, for an average cycle length of 28 days, the test and sampling had to be performed around day 20 of the cycle. Each participant was responsible for taking the loading substances, collecting the urine and saliva samples at home, and storing the samples at −20 °C. The following day, participants came to the research clinic at the NWU Centre of Excellence for Nutrition for collection of blood samples, and the determination of blood pressure, temperature, BMI (weight and height), and body fat percentage. Venous blood samples were drawn by a medical professional into EDTA and serum tubes (Becton Dickinson) via antecubital venipuncture. Completed questionnaires were also collected on this day. In a follow-up inquiry via an electronic form where participants could indicate more than one option, information was gathered about the reason why the participants were using a COC at the time. We received 20 out of 24 responses which were as follows: Contraception, 12/20; Period regulation, 12/20; Acne treatment, 7/20; Menstrual cramp treatment, 6/20; Polycystic ovary syndrome (PCOS) treatment, 3/20; Endometriosis treatment, 1/20; Other, 1/20.

### 2.2. Dietary Evaluation

The food frequency questionnaire was set up by a research dietician at the Centre of Excellence for Nutrition, North-West University, Potchefstroom Campus. The food frequency questionnaire was developed specifically to include foods that could have an effect on biotransformation, by consulting the literature. The questionnaire is not quantified and only the frequency of intake in the week prior to the test was assessed. The frequency per week was converted to an average frequency per day for statistical analysis. The questionnaire was self-administered by the participants.

### 2.3. Medical Symptoms Questionnaire

An openly accessible Medical Symptoms Questionnaire (MSQ, File S1 included in the Supplementary Material online) from the Departments of Medicine, Mercy Hospital and Maine Medical Center, Portland [31] was completed by each participant. The MSQ contains 19 domains, most with 4 items, totaling 75 indicators to each of which the patient assigns a Likert scale numerical ranking (0 being “never” or “seldom” and 4 being “frequently with severe effect”). The 75 questions are divided into 15 main sections. The scores were calculated as the mean of the items per section and the items were also combined in an overall MSQ score. The reliability (Cronbach’s alpha value) of the questionnaire had been confirmed previously for a South African Caucasian population [30].

### 2.4. Piper Fatigue Scale Assessment

Each participant also completed an English version of the revised Piper Fatigue Scale [32] (PFS, File S2 included in the Supplementary Material online). The revised PFS used consisted of 22 numerical items which assessed fatigue experienced by the patients. All items were coded on a 0–10 numeric scale. The revised PFS measured four dimensions of subjective fatigue: behavior/severity, affective meaning, sensory, and cognitive/mood. The reliability (Cronbach’s alpha value) of the questionnaire had been confirmed previously for a South African Caucasian population [30]. To calculate a total fatigue score, the cumulative sum of all was divided by the total number of items. According to the scoring instructions of the PFS, the severity of the fatigue can be classified according to the following severity codes: None (0), Mild (1–3), Moderate (4–6), and Severe (7–10).

### 2.5. Biomedical/Physiological Measurements

After blood sample collection, blood pressure and body temperature were measured. Systolic and diastolic blood pressure was measured while the participant was lying down, using an aneroid sphygmomanometer. Body temperature was measured using the tympanic method. Weight and height were measured with a calibrated digital scale with a stadiometer (Seca 264, Hamburg, Germany), with the subjects standing upright with their heads in the Frankfort plane according to the standards of the International Society for the Advancement of Kinanthropometry (ISAK). Body mass index (BMI) was calculated as weight divided by height squared. Body composition (fat percentage) was evaluated using air displacement plethysmography (ADP) and the BODPOD scale (Life Measurement, Inc, Concord, CA, USA) according to manufacturer’s instructions and recommendations. ADP is considered a reliable method for the measurement of body composition [33,34]. Participants were wearing tight fitting clothes and a swim cap.

### 2.6. Reagents

Caffeine, allopurinol, acetaminophen-glucuronide, acetaminophen-sulphate, high purity trifluoroacetic acid, salicyluric acid, 3-phenyl butyric acid, N,N-diethyl-paraphenylenediamine (DEPPD), sodium acetate, ammonium acetate, hydrogen peroxide, 2-acetamidophenol, 2,3 and 2,5 dihydroxy benzoic acid, catechol, N,O Bis(trimethylsilyl)fluoro acetamide (BSTFA), trimethylchlorosilane (TMCS), hydrochloric acid, sodium sulfate, pyridine, 2,4,6-tris-2-(pyridyl)- 1,3,5-triazine (TPTZ), Ferri Chloride (FeCl_3_·6H_2_O), and metaphosphoric acid (MPA) were all obtained from Sigma-Aldrich (now Merck). Acetaminophen-mercapturate was supplied by Toronto Research Chemicals. HPLC grade acetonitrile, methanol, and water was supplied by Burdick & Jackson. Ferrous sulphate was supplied by Labchem. Ethyl acetate and diethyl ether, formic acid, acetic acid, and butanoic HCL were purchased from Merck. Carnitine and acylcarnitine reference standards and deuterated analogues were obtained from Cambridge Isotope Laboratories. GSH/GSSG-412 assay kits were purchased from Aoxre Bio-Sciences.

### 2.7. Biotransformation Efficiency

Biotransformation analyses was performed exactly as described in detail in Erasmus et al. [30]. In brief, phase I (CYP1A2) biotransformation activity was measured by HPLC through a caffeine clearance test on saliva samples that were collected two and eight hours after taking 150 mg caffeine (Regmakers^®^) at 08:00 in the morning. Phase II biotransformation (sulfate, glucuronide, glycine, and glutathione conjugation) was assessed by measuring the levels of acetaminophen-sulfate, acetaminophen-glucuronide, acetaminophen-mercapturic acid, and salicyloyl-glycine (salicyluric acid) by HPLC on overnight urine samples, collected after taking 600 mg aspirin (Disprin^®^) and 1000 mg acetaminophen (Panado^®^) at 21:00 in the evening.

### 2.8. Secondary Products from the Acetylsalicylic Acid Challenge

The three secondary products from the acetylsalicylic acid challenge—catechol, 2,3-DHBA, and 2,5-DHBA—were analyzed using GC-MS. Spontaneous hydroxylation of salicylic acid by hydroxyl radicals results in the formation of 2,3-DHBA and catechol. The levels of these metabolites, therefore, give an indication of the level of hydroxyl radicals present. 2,5-DHBA is produced through enzymatic conversion of salicylic acid by CYP2E1 and CYP3A4 and its levels may give an indication of the activity of these enzymes.

### 2.9. Additional Biochemical Measurements

Total free and acyl-carnitines were quantified as described in Erasmus et al. [30]. The role of carnitines in the production of energy (acetylcarnitine), and in biotransformation of accumulated xenobiotic carboxylic acids and acyl groups, made it a good supplementary analysis. Both urine creatinine and uric acid values were determined using the creatinine (enzymatic) and uric acid (AOX) kits on the Indico Clinical Chemistry Analyzer.

### 2.10. Serum Peroxides

Based on the method described by Verde et al. [35], total serum peroxides (marker of ROS) were measured spectrophotometrically by monitoring the kinetic FeSO_4_-dependent formation of the N,N-diethyl-paraphenylenediamine (DEPPD) cation as described in Erasmus et al. [30]. Samples were analyzed in triplicate and measurements expressed as units (1 unit equaling 1 mg H_2_O_2_/L).

### 2.11. Total Glutathione

Total glutathione levels in blood were determined using the GSH/GSSG kit from Aoxre Bio-Science according to the kit protocol, with minor adjustments to allow the use of smaller blood volumes as described in Erasmus et al. (2019) [30]. Samples were analyzed in triplicate.

### 2.12. Ferric Reducing Ability of Plasma (FRAP)

FRAP assays were performed on a BIO-TEK Powerwave^TM^ XS2 microplate spectrophotometer to measure the reduction of ferric 2,4,6-tris-2-(pyridyl)- 1,3,5-triazine (TPTZ) to ferrous TPTZ, according to [36] and as described in detail in Erasmus et al. (2019) [30]. Samples were analyzed in triplicate and measurements expressed as units (1 unit equaling 1 μmol FeSO_4_/L).

### 2.13. Statistical Analyses

The Hotelling T-squared statistic, derived from a principal component analysis (PCA) model for each group separately, was used on the entire data set to identify outlying samples. Based on the result from this test, no participants were excluded. Therefore, for controls *n* = 25, whereas for COC users *n* = 24. Given that no separation was observed between the two COC dose groups after performing a PCA on each data set (Supplementary Figure S1 online), and considering the limited sample size, all COC users were combined in one group. Biotransformation, serum peroxide, and antioxidant capacity data were log transformed, whereas basic characteristic data, in addition to questionnaire data, were analyzed without transformation. Data sets were complete, i.e., there were no missing data. Parametric univariate analyses were employed and included the independent samples t-test and its associated effect size (ES, Cohen’s d-value) using MATLAB 2010, version 7.10.0 (R2010a; Natick, MA, USA: The MathWorks Inc). Differences between groups were considered to be of practical significance when d ≥ 0.5. Correction for multiple testing was done using the Benjamini and Hochberg adjustment to control the rate of false discovery (BH FDR) [37]. Because small effects were expected and the sample size of the study population was small, a false discovery rate of 10% was considered acceptable. Therefore, an adjusted *p*-value ≤ 0.1 was considered statistically significant. Graphs were constructed using GraphPad Prism 8.

## 3. Results

### 3.1. Baseline Characteristics of Study Population

The baseline characteristics of the two groups were comparable (Table 1), although COC users tended to have a slightly elevated blood pressure (BP). This effect was of practical significance for both systolic and diastolic BP (ES = 0.57 and 0.52, respectively). One COC user appeared to be hypertensive (BP = 130/80).

### 3.2. Dietary Evaluation

**Figure 1:**
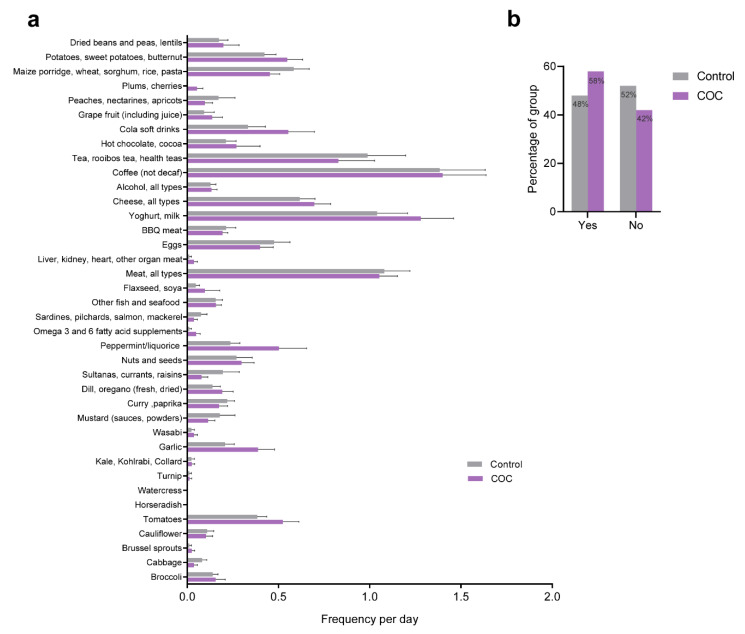
Dietary evaluation and nutritional supplement use. (**a**) The consumption frequency of specific food items per week was converted to an average frequency per day for statistical analysis. No correlations were found between any of the food items listed and either of the groups. (**b**) Proportion of participants that used nutritional supplements per group.

Because diet can directly influence health and wellbeing, and the activity and expression of many of the enzymes involved in biotransformation are regulated or influenced by dietary components, we assessed the diets of all participants with regard to food types that are known to influence the activity of the phase II enzymes, in particular. The results from the food frequency questionnaire are given in Figure 1a. In addition, we recorded the use of nutritional supplements (yes/no) among participants. Overall, no differences were found between COC users and controls with regard to the food types assessed. The use of nutritional supplements or vitamins appeared to be slightly higher in COC users (Figure 1b).

### 3.3. Health Status and Wellbeing

**Figure 2:**
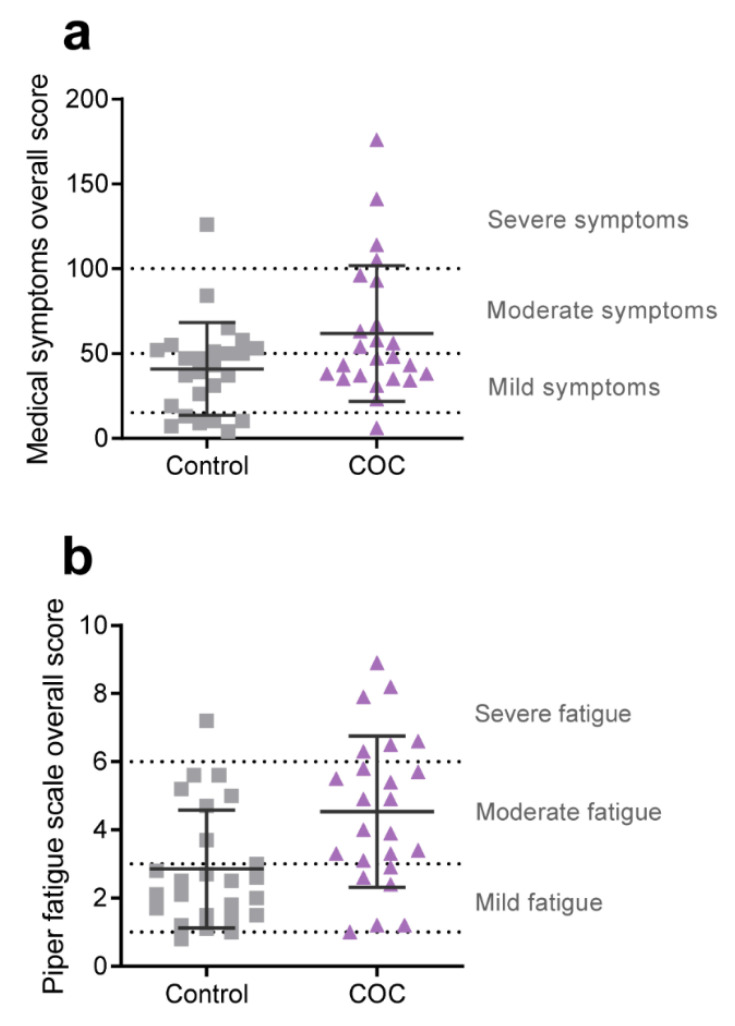
Overall scores for the (**a**) medical symptoms questionnaire and the (**b**) Piper fatigue scale. Dashed lines indicate the cut-off points of the severity levels listed on the right of each graph (as generally applied for the MSQ by physicians affiliated with the Institute for Functional Medicine [38,39] and described in Piper et al. [40] for the PFS). Squares and triangles represent individual controls and COC users, respectively. Horizontal solid lines and error bars indicate means ± SD.

To determine how the established use of COCs containing DRSP/EE affects general health and wellbeing, we quantified the incidence and frequency of various medical symptoms, and the level of fatigue, experienced by all the participants. The questions included in the medical symptoms questionnaire (MSQ) can be divided into 15 categories: head, ears, eyes, skin, nose heart, emotions, mind, digestive tract, mouth, lung, energy, weight, joint, and other. Quantification of the MSQ data indicated that, overall, COC users experienced a higher incidence of medical symptoms (ES = 0.52; Table 2 and Figure 2a). Specifically, COC users more often complained about weight-related issues (binge eating, cravings, compulsive eating, over-/underweight, water retention; ES = 0.73) and symptoms relating to the nose (stuffy nose, sinus problems, hay fever, sneezing attacks, excessive mucus; ES = 0.62) (Table 2). Furthermore, COC users tended to experience more problems relating to the digestive tract (nausea/vomiting, diarrhea, constipation, bloating, belching, heartburn, stomach pain; ES = 0.43). The Piper fatigue scale was used to assess the level of fatigue experienced by each participant and was quantified to calculate an overall fatigue score and to determine on what level the participants were experiencing the fatigue. From Figure 2b it is clear that COC users were suffering from moderate to severe fatigue compared to controls who were exhibiting only mild fatigue (PFS total score ES = 0.76; Table 3). Further analysis indicated that all subscales were significantly affected (Table 3). The fatigue experienced by COC users most significantly hampered their ability to work, socialize, and engage in pleasant activities, and was experienced as having a destructive/negative impact on them. Combined, these data indicate that COC users in this study generally felt less healthy and markedly more tired compared to healthy controls who did not use any hormonal products.

### 3.4. Biotransformation Efficiency

**Figure 3:**
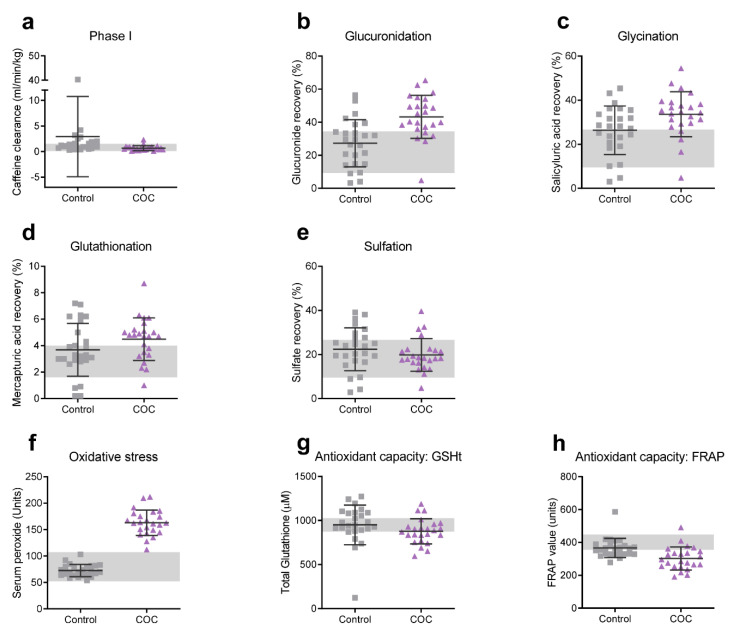
Untransformed data of phase I and II biotransformation efficiency, serum peroxides, and antioxidant capacity. (**a**) Phase I (CYP1A2) activity was measured as the caffeine clearance rate in saliva. Phase II (conjugation) efficiency was assessed by measuring the recovery of (**b**) APAP glucuronide, (**c**) salicyluric acid, (**d**) APAP mercapturic acid, and (**e**) APAP sulfate in urine. (**f**) Serum peroxides were measured as an indicator of oxidative stress and (**g**) total red blood cell glutathione and (**h**) ferric reducing ability of plasma as indicators of antioxidant capacity. Squares and triangles represent individual controls and COC users, respectively. Horizontal lines and error bars indicate means ± SD. Shaded background indicates reference ranges obtained by our own laboratory from a South African Caucasian group of 46 healthy individuals between the age of 18 and 35 years (22 men and 24 women).

After oral ingestion, the synthetic hormones contained in COCs are absorbed and undergo extensive first pass metabolism in the gut and liver and, for EE specifically, only a fraction (20–65%) of the original dose is directly bioavailable [28]. Metabolism of these hormones involves both phase I and phase II biotransformation reactions. Daily intake of synthetic hormones may, therefore, increase the total toxic load of the biotransformation system. Moreover, EE containing COCs have been implicated in multiple pharmacokinetic drug interactions [28]. Several studies have suggested that overall health and wellbeing is associated with biotransformation efficiency, and several diseases have been linked with imbalanced phase I and II biotransformation reactions (reviewed in Liska et al. [29]). We were, therefore, curious to know how overall liver biotransformation efficiency was affected in COC users compared to controls. One of the most abundant CYP450 enzymes involved in phase I biotransformation activity in the liver is the CYP1A2 enzyme [41,42]. Functional analysis of CYP1A2 activity was undertaken by measuring caffeine clearance in the saliva of the participants. The results in Table 4 and Figure 3a show that CYP1A2 activity was significantly reduced in COC users compared to controls (ES = 0.63). This is in agreement with previous studies on other EE-containing COC formulations [43,44].

Metabolites from phase I biotransformation can subsequently undergo phase II conjugation reactions in order to increase the water solubility and facilitate the excretion of these metabolites. Phase II conjugation reactions include methylation, acetylation, glutathione conjugation, glucuronidation, sulfation, amino acid (particularly glycine) conjugation, and carnitine conjugation. In order to assess phase II biotransformation activity, the production of certain conjugated metabolites of the probe substances were analyzed. Glucuronidation of acetaminophen (APAP; paracetamol) and glycination of acetylsalicylic acid (aspirin) were significantly upregulated in COC users (ES = 0.81 and 0.49, respectively; Table 4 and Figure 3b,c). GSH conjugation of the APAP metabolite N-acetyl-p-benzoquinone imine (NAPQI) also appeared to be higher in COC users (ES = 0.42), whereas APAP-sulfation did not seem to be affected (Table 4 and Figure 3d,e). Finally, the level of urinary acylcarnitines was slightly elevated in COC users (ES = 0.41; Table 4). Taken together, COC use clearly alters the biotransformation homeostasis in young women.

### 3.5. Serum Peroxides and Antioxidant Capacity

Oxidative stress is tightly linked to biotransformation activity: phase I reactions can contribute to the production of oxidants, whereas phase II enzyme activities are highly inducible by ROS via the Nrf2-Keap1 pathway and aid in the detoxification of reactive metabolites [45]. Imbalances in the biotransformation pathway due to increased toxic load or insufficient enzyme activity can result in the production of reactive oxygen species or metabolites (ROS/ROM). Excessive production of ROS/ROM may result in oxidative stress which may, in turn, negatively affect physical and mental health [46,47]. Furthermore, previous studies have strongly correlated COC use (including those containing DRSP/EE [21]) with oxidative stress. In order to confirm this in our study, we measured serum peroxide levels and secondary products from the acetylsalicylic acid challenge (catechol and 2,3-DHBA) in all COC users and controls. Our results showed that COC users indeed had markedly elevated serum peroxide levels (162.89 ± 24.00 Units) compared to controls (72.56 ± 11.52 Units) (ES = 5.26; Table 4, Table S1 and Figure 3f). No difference was observed in the production of 2,3-DHBA and catechol levels between COC users and controls.

Elevated ROS levels only cause oxidative stress if the production of oxidants exceeds the antioxidant capacity of the body. Glutathione (GSH) is one of the most prominent endogenous antioxidants. It can be oxidized to GSSG by GSH peroxidase (GPx) in the presence of hydroperoxides and be recycled through GSH reductase. We measured total red blood cell GSH and found that these levels were lower but not significantly reduced in COC users (Table 4 and Figure 3g). However, as an additional measure of the antioxidant capacity, we compared the ferric reducing ability of plasma (FRAP) and found that FRAP in COC users was significantly lower (ES = 0.91; Table 4 and Figure 3h) than in controls.

## 4. Discussion

This is the first study assessing the impact of COCs containing DRSP/EE on the general subjective health status and the homeostasis between phase I and II liver biotransformation reactions in young women between the ages of 18 and 35. The baseline characteristics of controls and COC users in our study were comparable. COC use did not cause an increase in body weight, in agreement with previous studies [16,17,48]. Body fat percentage was also not affected. However, in contrast with other studies [49,50] a practical significant effect was observed on blood pressure. Analysis of the medical symptoms’ questionnaire data indicated that, overall, COC users tended to experience more health-related symptoms compared to healthy controls who do not use any hormonal products. A further striking observation from our study was that COC users suffered significantly more from fatigue, which negatively impacted their physical and social functioning. The MSQ and PFS results are in stark contrast with numerous other studies that have shown that the DRSP/EE combination of COCs has a beneficial effect with regard to general psychological and physical wellbeing [51,52,53,54,55]. A major difference between our study and former studies lies in the study design: the former studies were experimental in nature and conducted from the initiation of COC use up to six months or maximally two years. This means that the beneficial effects that were reported in these studies were observed within the first two years of COC use. No data is available on the effects beyond two years of COC use. Our study, by comparison, was observational and included women who were already using the COC for at least three months and up to 12 years when they participated in the study. Most women (65%) had used the COC for more than two years (average 46.0 ± 34.8 months). Moreover, in the former studies women may have requested to use COCs due to menstrual problems they experienced. In that context, the pill would most likely have had a positive effect. In our study, women were already using the pill (mostly for contraception and cycle regulation) and were asked to evaluate their current state of health and wellbeing and not to compare it with a ‘before’ situation. Although different instruments were used to evaluate physical and psychological wellbeing in previous studies, our results indicate that COCs containing DRSP/EE adversely affect the subjective health status and cause moderate to severe fatigue in healthy young women.

Both a disturbed biotransformation homeostasis and oxidative stress have been linked to various health problems and disease states [29,56]. In COC users specifically, oxidative stress has been associated with increased high-sensitivity C-reactive protein (hsCRP), a marker of low-grade chronic inflammation [57]. In line with numerous other studies, we report here the occurrence of oxidative stress with COC use. In agreement with de Groote et al. [21], serum peroxide levels were significantly higher in women using COCs containing DRSP/EE. Because no difference was observed in the production of 2,3-DHBA and catechol (secondary metabolites of salicylic acid metabolism), it appears as if hydroxyl radicals do not significantly contribute to the induction of oxidative stress in COC users. The exact nature and origin of the ROS we measured are, therefore, not known and should be further investigated. Nevertheless, because plasma antioxidant capacity was also significantly reduced in COC users, our results suggest that these women are exposed to chronic oxidative stress.

Our data further showed that phase I and II biotransformation activities were altered in young women using COCs containing DRSP/EE. In general, a strong link exists between biotransformation and oxidative stress [29]: when the production rate of oxidants from phase I reactions exceeds the rate of efficient neutralization by phase II enzymes or the antioxidant defense system of the body, this may be a potential source of ROS. However, this explanation is not supported by our data, which showed that phase I activity, measured as caffeine clearance via the CYP1A2 enzyme, was significantly reduced in COC users (in agreement with a former study by Rasmussen et al. [58]), whereas phase II activity appeared to be upregulated. We speculate that, rather than being a result of imbalanced biotransformation, the COC-induced ROS modulate biotransformation activity (for example by activating phase II enzymes such as UDP-glucuronosyl transferase (UGT) via the Nrf2-Keap1 pathway [59,60]) and other physiological processes that may negatively impact the health status of young women (such as the regulation of blood pressure [61,62]).

CYP1A2 is relatively highly expressed in the liver and plays a prominent role in drug metabolism [42]. EE is known to cause reversible inhibition in CYP1A2 [26], although there is some evidence indicating that CYP enzymes can be also inhibited by ROS such as hydrogen peroxide (H_2_O_2_) [63]. Reduced CYP1A2 activity may result in the prolonged exposure/increased circulation time of other substances that are metabolized by this enzyme, including drugs (e.g., acetaminophen), dietary flavonoids, and endobiotics (e.g., endogenous arachidonic acid, prostaglandins, estrogens). Several other CYPs (e.g., CYP2C19 and CYP2C9) are also inhibited by oral contraceptives [42]. Furthermore, EE is not the only contributor to this inhibitory effect: DRSP is also capable of inhibiting CYP enzymes CYP1A1, CYP2C9, CYP2C19, and CYP3A4 [26]. This broad inhibitory effect of phase I biotransformation may lead to increased effects of pharmacologically active drugs and a risk for overdosing, while the effect of prodrugs may be reduced.

With regard to phase II reactions, COC use significantly increased the glucuronic acid conjugation and glycine conjugation of the administered probe substances in this study. The strongest effect was observed on glucuronidation of APAP. Diet and nutritional supplements can influence the activity and expression of the phase II enzymes. However, because no differences were found in the consumption frequency of foods that are known to regulate phase II biotransformation, we concluded that the induction of phase II reactions was a result of COC use. Glucuronidation appears to be the preferred conjugation pathway involved in the metabolism of EE [64] and largely occurs in the gastrointestinal track [28]. DRSP metabolites also undergo glucuronidation [65]. The main enzymes involved in glucuronidation of APAP are UGT1A1 and UGT1A9 [66]. UGT1A1 is also responsible for glucuronidation of EE [67]. The expression of UGT is regulated by several transcription factors, including Nrf2 and the estrogen receptor (ER) [67]. In line with this, the UGT1A6 isoform is induced by ROS and EE in rat astrocytes [68] and mouse uterus [69], respectively. Therefore, the increase in UGT activity observed in the COC group may be mediated by COC-induced ROS signaling and/or signaling via the estrogen receptor. Increased UGT activity may result in increased energy expenditure towards detoxification because this reaction requires a high energy sugar (UDP-glucuronic acid) to facilitate conjugation to EE. This could put other ATP-dependent biochemical reactions and physiological processes under pressure and may contribute to the higher levels of fatigue noted in the COC users.

Amino acid (mainly glycine) conjugation is another mechanism for the elimination of xenobiotics and endobiotics from the body. The xenobiotic or endobiotic is first activated by ATP and conjugated to coenzyme A (CoASH) before undergoing amino acid conjugation in the mitochondria. The enzymes involved in the glycine conjugation of acetylsalicylic acid are acyl-CoA synthetase medium-chain (HXM-A; coded by the *ACSM2B* gene) and glycine *N*-acyltransferase (GLYAT). The increased formation of salicyluric acid (glycine conjugate of salicylic acid) in our study may, therefore, be the result of induction of either of the two enzymes. Scientific reports on the effect of hormones or ROS on the expression or regulation of HXM-A are limited. GLYAT, however, has been shown to be regulated by estrogens [70]. In mice receiving EE via oral gavage, GLYAT expression was increased in uterine tissue [69]. Increased production of salicyluric acid in the COC users in our study may, therefore, be a result of EE-induced GLYAT expression. Increased GLYAT activity will lead to increased glycine consumption. Interestingly, serum glycine levels are reduced in DRSP/EE COC users [71]. This may indicate that glycine availability is limited in COC users, which in turn may have biochemical consequences. Firstly, limited glycine availability will inhibit the glycination of other (xenobiotic) acyl-CoAs and, thus, the release of CoASH. If the acyl-CoA is not hydrolyzed by acyl-CoA hydrolases, this will in turn lead to the sequestration of CoASH and the inhibition of mitochondrial metabolic processes, such as β-oxidation of fatty acids. Furthermore, xenobiotic acyl-CoAs may inhibit certain enzymes and acetylate protein thiol groups, or they can be excreted as acylcarnitines [72]. Our data indicate that COC users tended to excrete more acylcarnitines (ES = 0.41, Table 4). Although the effect was not statistically significant, it may be an early sign that the glycine conjugation system is being put under pressure. It should also be recognized that increased urinary acylcarnitines may be an additional consequence of a disturbed redox balance (NAD^+^/NADH ratio), which may result in reduced β-oxidation of fatty acids. A second consequence of limited glycine availability could be the limited synthesis of other important molecules, including glutathione (GSH), which plays an important role as an antioxidant and as a conjugation moiety in phase II reactions catalyzed by GSH S-transferase (GST). Although total red blood cell GSH levels (i.e., GSH + GSSG) tended to be lower in COC users in our study, the effect was not significant. However, the high levels of serum peroxides will inevitably have led to increased oxidation of GSH to GSSG. This, together with potential insufficient biosynthesis (due to glycine shortage), may have limited the availability of GSH for conjugation, and may have prevented significant upregulation of the GST reaction in our study.

## 5. Conclusions

Our data show that overall health status is adversely affected, and phase I and II biotransformation activity altered, in young women using COCs containing DRSP/EE. COCs containing DRSP/EE, therefore, seem to disturb the biotransformation homeostasis in young women by upregulating phase II reactions and downregulating phase I reactions. This may have negative consequences, because essential co-factors (e.g., GSH, CoASH) may become limited or even depleted, which will result in the inhibition of various metabolic processes (e.g., β-oxidation, redox regulation, detoxification). In turn, this may negatively affect the production of other macromolecules and ATP, and disrupt regular biochemical processes. In conjunction with increased energy expenditure on phase II detoxification reactions, this may limit energy availability, possibly contributing to the higher levels of fatigue and reduced health status reported by the COC users. We also confirmed that the COC users had a higher oxidative stress status than controls. Considering the findings from the current study, together with those included in the discussion, we speculate that the oxidative stress induced by COCs may contribute significantly to the detrimental effects observed in, or experienced by, COC users. Further investigation should confirm whether this is the case and shed light on the molecular mechanisms involved. Although the sample size was sufficient to reach significance for most parameters that were assessed, a larger sample size would have yielded more power and clearer differentiation between the groups. Our study focused on COCs containing DRSP/EE; however, other studies indicate that other COC formulations containing EE may have the same effects on biotransformation efficiency [28,43,44]. In order to identify safer alternatives for contraception or alleviation of menstrual symptoms, the effect of these formulations and other forms of contraceptives (e.g., injections, implants, IUDs, and contraceptive pills containing E2 instead of EE) on biotransformation homeostasis and health status should also be determined. From this and other studies, it is evident that COCs have, in addition to beneficial properties, potential harmful effects due to long-term use, and users should be aware of all of these consequences in order to make an informed choice with regard to contraceptive type.

## Figures and Tables

**Table 1:** Baseline characteristics of study population. ES, effect size. Cohen’s d value: 0.2, small effect; 0.5, medium effect; 0.8, large effect; 1.3, very large effect.

	Control		COC		Control vs. COC	
	Mean	(SD)	Mean	(SD)	ES (Cohen’s d)	BH FDR Adjusted *p*-Value
Weight (kg)	64.58	(9.27)	67.49	(12.84)	0.23	0.528
Height (m)	1.68	(0.06)	1.69	(0.08)	0.16	0.528
BMI (kg/m^2^)	22.81	(2.42)	23.44	(3.42)	0.18	0.528
Age (years)	24.96	(4.71)	23.38	(2.89)	0.34	0.377
Systolic BP (mmHg)	107.52	(7.10)	111.54	(6.45)	0.57	0.178
Diastolic BP (mmHg)	68.96	(6.46)	72.29	(5.12)	0.52	0.178
Body fat %	28.79	(5.54)	30.41	(7.72)	0.21	0.528
COC Duration (months)	N/A		46.0	(34.8)		
(range)			(3–144)			
Proportion smokers (<5 cigarettes/day)	12%		4%			

**Table 2:** Results from the Medical Symptoms Questionnaire (MSQ). SD, Standard deviation. ES, effect size. Cohen’s d value: 0.2, small effect; 0.5, medium effect; 0.8, large effect; 1.3, very large effect. * The BH FDR adjusted *p*-value was considered significant when ≤0.1.

	Control		COC		Control vs. COC	
Sub-Scale	Mean	(SD)	Mean	(SD)	ES (Cohen’s d)	BH FDR Adjusted *p*-Value
Overall Score	40.92	(27.26)	61.71	(40.01)	0.52	0.216
Head	3.28	(2.46)	4.29	(2.94)	0.34	0.380
Ears	1.24	(1.96)	1.83	(2.10)	0.28	0.455
Eyes	2.32	(2.06)	2.88	(2.47)	0.22	0.490
Skin	3.40	(3.30)	4.33	(4.03)	0.23	0.490
Nose	3.80	(3.44)	7.29	(5.67)	0.62	0.108
Heart	0.96	(1.34)	2.13	(3.49)	0.33	0.337
Emotions	4.44	(3.12)	6.08	(4.51)	0.36	0.337
Mind	5.04	(4.89)	5.63	(4.52)	0.12	0.666
Digestive Track	4.04	(3.70)	7.25	(7.46)	0.43	0.266
Other	1.24	(1.83)	2.04	(3.20)	0.25	0.455
Mouth/Throat	1.28	(1.95)	1.71	(3.06)	0.14	0.601
Lungs	0.52	(1.26)	0.79	(1.72)	0.16	0.601
Energy/Activity	3.16	(2.76)	4.54	(3.46)	0.40	0.337
Weight	3.64	(3.35)	7.25	(4.93)	0.73	0.077 *
Joint/Muscle	2.56	(2.47)	3.67	(3.55)	0.31	0.380

**Table 3:** Results from the Piper Fatigue Scale (PFS). The Behavioral/Severity subscale measured the impact fatigue might have had on activities of daily living; the Affective Meaning subscale determined the emotional meaning attributed to fatigue; the Sensory subscale analyzed the mental, physical, and emotional symptoms of fatigue; the Cognitive/Mood subscale analyzed the effect fatigue might have had on cognitive function and mood. SD, Standard deviation. ES, effect size. Cohen’s d value: 0.2, small effect; 0.5, medium effect; 0.8, large effect; 1.3, very large effect. * The BH FDR adjusted *p*-value was considered significant when ≤0.1.

	Control		COC		Control vs. COC	
Sub-Scale	Mean	(SD)	Mean	(SD)	ES Cohen’s d	BH FDR Adjusted *p*-Value
Overall Score	2.86	(1.73)	4.54	(2.23)	0.76	0.009 *
Behavioral/Severity	2.19	(1.53)	4.22	(2.60)	0.78	0.005 *
Affective Meaning	2.52	(2.00)	4.70	(2.58)	0.85	0.005 *
Sensory	3.59	(2.13)	4.90	(2.29)	0.57	0.044 *
Cognitive/Mood	3.13	(1.74)	4.34	(2.15)	0.56	0.044 *

**Table 4:** Log transformed data of biotransformation efficiency, serum peroxides, and antioxidant capacity. Raw data was log transformed to apply parametric statistical analyses. SD, Standard deviation. ES, effect size. Cohen’s d value: 0.2, small effect; 0.5, medium effect; 0.8, large effect; 1.3, very large effect. * The BH FDR adjusted *p*-value was considered significant when ≤0.1. Untransformed data is available in Supplementary Table S1 online.

	Control		COC		Control vs. COC	
Variable	Mean	(SD)	Mean	(SD)	ES (Cohen’s d)	BH FDR Adjusted *p*-Value
Creatinine	2.28	(0.59)	2.74	(0.56)	0.78	0.021 *
Uric acid	0.81	(0.36)	0.86	(0.46)	0.10	0.745
Uric acid:Creatinine ratio	0.16	(0.11)	0.10	(0.05)	0.57	0.036 *
Caffeine clearance (Phase I)	0.91	(0.69)	0.47	(0.26)	0.63	0.020 *
APAP-glucuronide	3.17	(0.68)	3.72	(0.47)	0.81	0.008 *
APAP-sulfate	3.03	(0.58)	2.97	(0.38)	0.10	0.745
APAP-mercapturic acid	1.43	(0.54)	1.66	(0.33)	0.42	0.145
Salicyluric acid	3.18	(0.60)	3.48	(0.44)	0.49	0.107
Catechol	2.39	(0.76)	2.18	(0.65)	0.29	0.412
2,3-DHBA	1.41	(0.64)	1.26	(0.57)	0.23	0.525
2,5-DHBA	3.85	(0.85)	3.97	(0.98)	0.12	0.745
Carnitine, Total-Free	0.91	(0.35)	0.90	(0.46)	0.01	0.981
Acyl-Carnitine, Total	1.10	(0.44)	1.28	(0.44)	0.41	0.253
Acyl-Carnitine:Free Carnitine ratio	0.94	(0.53)	1.16	(0.65)	0.35	0.287
PhaseI:PhaseII ratio (Sulfation)	2.07	(0.92)	1.42	(0.73)	0.70	0.023 *
PhaseI:PhaseII ratio (Glycination)	1.94	(0.93)	1.06	(0.65)	0.94	0.003 *
PhaseI:PhaseII ratio (Glucuronidation)	1.96	(1.04)	0.94	(0.64)	0.98	0.002 *
Serum Peroxides	4.29	(0.15)	5.09	(0.15)	5.26	<0.001 *
FRAP	5.90	(0.14)	5.69	(0.23)	0.91	0.003 *
GSHt	6.80	(0.44)	6.77	(0.16)	0.08	0.745

## Data Availability

The datasets generated and analyzed during the current study are available through the North-West University’s institutional data repository DaYta Ya Rona (Figshare) via the following DOI:10.25388/nwu.15123570.

## Supplementary Materials

The following supplementary material is available online at www.mdpi.com/article/10.3390/ijerph182010607/s1. File S1: Medical Symptoms Questionnaire. File S2: Piper Fatigue Scale. Figure S1: PCA plots of 3mg DRSP/30μg EE vs. 3 mg DRSP/20 μg EE. Table S1: Untransformed data of biotransformation efficiency, serum peroxide levels, and antioxidant capacity.
