# Supplementary material for: Health Status Is Affected, and Phase I/II Biotransformation Activity Altered in Young Women Using Oral Contraceptives Containing Drospirenone/Ethinyl Estradiol
Source: Int J Environ Res Public Health. 2021 Oct 10;18(20):10607. doi: 10.3390/ijerph182010607 (PMC8535641; doi:10.3390/ijerph182010607)
Supplement: Supplementary file 1 [file ijerph-18-10607-s001.zip › ijerph-1382359-supplementary.pdf]

Supplementary Material

Health Status Is Affected, and Phase I/II Biotransformation Activity Altered in Young Women Using Oral Contraceptives Containing Drospirenone/Ethinyl Estradiol

Gerda Venter *, Carien L. van der Berg, Francois H. van der Westhuizen and Elardus Erasmus *

Human Metabolomics, Faculty of Natural and Agricultural Sciences, North-West University (Potchefstroom Campus), 11 Hoffman Street, Potchefstroom 2531, South Africa; carien.vanderberg@nwu.ac.za (C.L.v.d.B.); francois.vanderwesthuizen@nwu.ac.za (F.H.v.d.W.)
* Correspondence: gerda.venter@nwu.ac.za (G.V.); lardus.erasmus@nwu.ac.za (E.E.); Tel.: +27-18-299-1867 (G.V.); +27-18-299-2305 (E.E.)

File S1

eBOSS study

Ethics number: NWU-00344-16-S1

MEDICAL SYMPTOMS QUESTIONNAIRE

NB!: POINT SCALE: **HOW OFTEN AND SEVERELY DO YOU EXPERIENCE THE FOLLOWING SYMPTOMS - IN THE LAST 30 DAYS:**		Never / Almost never	Occasionally, effect is not severe	Occasionally, effect is severe	Frequently, effect not severe	Frequently, effect is severe	NB!: POINT SCALE: **HOW OFTEN AND SEVERELY DO YOU EXPERIENCE THE FOLLOWING SYMPTOMS - IN THE LAST 30 DAYS:**		Never / Almost never	Occasionally, effect is not severe	Occasionally, effect is severe	Frequently, effect not severe	Frequently, effect is severe
		**0**	**1**	**2**	**3**	**4**			**0**	**1**	**2**	**3**	**4**
**HEAD**	Headaches	0	1	2	3	4	**EYES**	Watery or itchy eyes	0	1	2	3	4
	Faintness	0	1	2	3	4		Swollen, reddened or sticky eyelids	0	1	2	3	4
	Dizziness	0	1	2	3	4		Bags or dark circles under eyes	0	1	2	3	4
	Insomnia	0	1	2	3	4		Blurred or tunnel vision (not including near- or far-sightedness)	0	1	2	3	4
**EARS**	Itchy ears	0	1	2	3	4	**SKIN**	Acne	0	1	2	3	4
	Ear aches, ear infections	0	1	2	3	4		Hives, rashes, dry skin	0	1	2	3	4
	Drainage from ear	0	1	2	3	4		Hair loss	0	1	2	3	4
	Ringing in ears, hearing loss	0	1	2	3	4		Flushing, hot flashes	0	1	2	3	4
								Excessive sweating	0	1	2	3	4
**NOSE**	Stuffy nose	0	1	2	3	4	**MOUTH/ THROAT**	Chronic coughing	0	1	2	3	4
	Sinus problems	0	1	2	3	4		Gagging, frequent need to clear throat	0	1	2	3	4
	Hay fever	0	1	2	3	4		Sore throat, hoarseness, loss of voice	0	1	2	3	4
	Sneezing attacks	0	1	2	3	4		Swollen or discoloured tongue, gums, lips	0	1	2	3	4
	Excessive mucus formation	0	1	2	3	4		Cancer sores	0	1	2	3	4
**HEART**	Irregular or skipped heartbeat	0	1	2	3	4	**LUNGS**	Chest congestion	0	1	2	3	4
	Rapid or pounding heartbeat	0	1	2	3	4		Asthma, bronchitis	0	1	2	3	4
	Chest pain	0	1	2	3	4		Shortness of breath	0	1	2	3	4
	Excessive sweating	0	1	2	3	4		Difficulty breathing	0	1	2	3	4

		0	1	2	3	4
**EMO-TIONS**	Mood swings	0	1	2	3	4
	Depression	0	1	2	3	4
	Anger, irritability, aggressiveness	0	1	2	3	4
	Anxiety, fear, nervousness	0	1	2	3	4
**MIND**	Difficulty in making	0	1	2	3	4
	Stuttering or stammering	0	1	2	3	4
	Slurred speech	0	1	2	3	4
	Learning disabilities	0	1	2	3	4
	Poor memory	0	1	2	3	4
	Confusion, poor comprehension	0	1	2	3	4
	Poor concentration	0	1	2	3	4
	Poor physical coordination	0	1	2	3	4
**DIGES-TIVE TRACT**	Nausea, vomiting	0	1	2	3	4
	Diarrhea	0	1	2	3	4
	Constipation	0	1	2	3	4
	Bloated feeling	0	1	2	3	4
	Belching, passing excessive gas	0	1	2	3	4
	Heartburn	0	1	2	3	4
	Intestinal / stomach pain	0	1	2	3	4
**OTHER**	Frequent illness	0	1	2	3	4
	Frequent or urgent urination	0	1	2	3	4
	Genital itch or discharge	0	1	2	3	4
	Painful lymph nodes (cervical, axillary, inguinal, or supraclavicular)	0	1	2	3	4

		0	1	2	3	4
**ENERGY/ ACTI-VITY**	Fatigue, sluggishness	0	1	2	3	4
	Apathy, lethargy	0	1	2	3	4
	Hyperactivity	0	1	2	3	4
	Restlessness	0	1	2	3	4
	Fatigue worsens with exertion (vigorous action or effort), plus post-exertional malaise (feeling ill after exertion)	0	1	2	3	4
**WEIGHT**	Binge eating / drinking	0	1	2	3	4
	Craving certain foods	0	1	2	3	4
	Excessive weight	0	1	2	3	4
	Compulsive eating	0	1	2	3	4
	Water retention	0	1	2	3	4
	Underweight	0	1	2	3	4
**JOINT/ MUSCLE**	Pain or aches in joints	0	1	2	3	4
	Arthritis	0	1	2	3	4
	Stiffness or limitation of movement	0	1	2	3	4
	Pain or aches in muscles	0	1	2	3	4
	Feeling of weakness or tiredness	0	1	2	3	4
	Migratory arthralgia - Pain in different joints at different times as if pain is travelling	0	1	2	3	4

File S2

eBOSS study

Ethics number: NWU-00344-16-S1

REVISED PIPER FATIGUE SCALE												
Please answer the questions below using the scale measurement in the next column		Scale measurement	Please mark appropriate scale									
			1	2	3	4	5	6	7	8	9	10
1	How long have you been feeling fatigue? (Check one response only): 1 - not feeling fatigue, 2 - minutes, 3 - hours, 4- days, 5 - weeks, 6 months, 7 - other (please describe)		1	2	3	4	5	6	7			
2	To what degree is the fatigue you are feeling now causing you distress?	1 No distress<br>10 A great deal	1	2	3	4	5	6	7	8	9	10
3	To what degree is the fatigue you are feeling now interfering with your ability to complete your work or school activities?	1 Non<br>10 A great deal	1	2	3	4	5	6	7	8	9	10
4	To what degree is the fatigue you are feeling now interfering with your ability to socialise with your friends?	1 Non<br>10 A great deal	1	2	3	4	5	6	7	8	9	10
5	To what degree is the fatigue you are feeling now interfering with your ability to engage in sexual activity?	1 - Non<br>10 A great deal	1	2	3	4	5	6	7	8	9	10
6	Overall, how much is the fatigue which you are now experiencing interfering with your ability to engage in the kind of activities you enjoy doing?	1 Non<br>10 A great deal	1	2	3	4	5	6	7	8	9	10
7	How would you describe the degree of intensity or severity of the fatigue which you are experiencing now?	1 Mild<br>10 Severe	1	2	3	4	5	6	7	8	9	10
8	To what degree would you describe the fatigue you are experiencing now as being	1 Pleasant<br>10 Unpleasant	1	2	3	4	5	6	7	8	9	10
9	To what degree would you describe the fatigue you are experiencing now as being	1 Agreeable<br>10 Disagreeable	1	2	3	4	5	6	7	8	9	10
10	To what degree would you describe the fatigue you are experiencing now as being	1 Protective<br>10 Destructive	1	2	3	4	5	6	7	8	9	10
11	To what degree would you describe the fatigue you are experiencing now as being	1 Positive<br>10 Negative	1	2	3	4	5	6	7	8	9	10
12	To what degree would you describe the fatigue you are experiencing now as being	1 Normal<br>10 Abnormal	1	2	3	4	5	6	7	8	9	10
13	To what degree are you now feeling	1 Strong<br>10 Weak	1	2	3	4	5	6	7	8	9	10
14	To what degree are you now feeling	1 Awake<br>10 Sleepy	1	2	3	4	5	6	7	8	9	10
15	To what degree are you now feeling	1 Lively<br>10 Listless	1	2	3	4	5	6	7	8	9	10
16	To what degree are you now feeling	1 Refreshed<br>10 Tired	1	2	3	4	5	6	7	8	9	10
17	To what degree are you now feeling	1 Energetic<br>10 Unenergetic	1	2	3	4	5	6	7	8	9	10

PIPER FATIGUE SCALE												
Please answer the questions below using the scale measurement in the next column		Scale measurement	Please mark appropriate scale									
			1	2	3	4	5	6	7	8	9	10
18	To what degree are you now feeling	1 Patient<br>10 Impatient	1	2	3	4	5	6	7	8	9	10
19	To what degree are you now feeling	1 Relaxed<br>10 Stressed	1	2	3	4	5	6	7	8	9	10
20	To what degree are you now feeling	1 Exhilarated<br>10 Depressed	1	2	3	4	5	6	7	8	9	10
21	To what degree are you now feeling	1 Able to concen-trate<br>10 Unable to	1	2	3	4	5	6	7	8	9	10
22	To what degree are you now feeling	1 Able to remember<br>10 Unable to	1	2	3	4	5	6	7	8	9	10
23	To what degree are you now feeling	1 Able to think clearly<br>10 Unable to	1	2	3	4	5	6	7	8	9	10
24	Overall, what do you believe is most directly contributing to or causing your fatigue?											
25	Overall, the best thing you have found to relieve your fatigue is:											
26	Is there anything else you would like to add that would describe your fatigue better to us?											
27	Are you experiencing any other symptoms right now?											

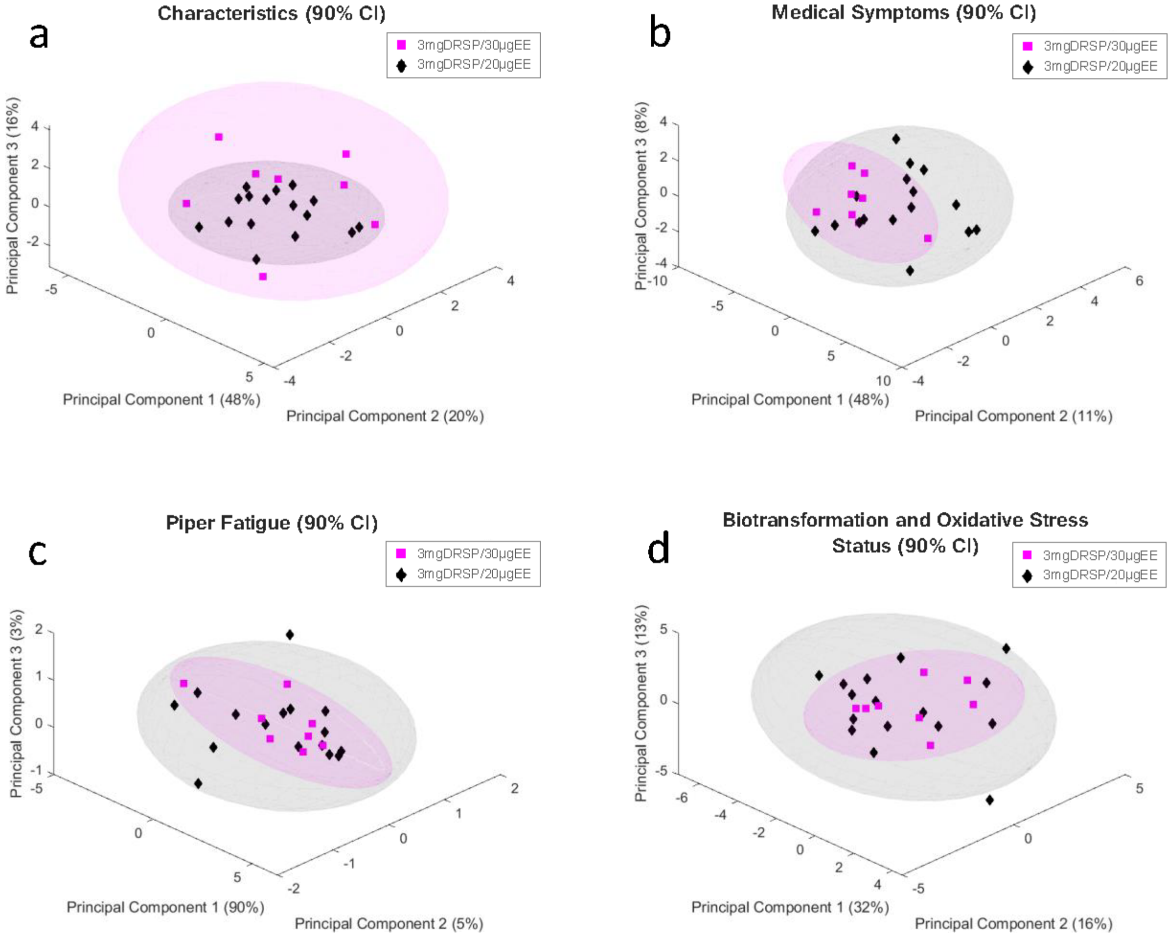

Figure S1 Three-dimensional principal component analysis (PCA) plots of 3mg DRSP/30µg EE vs 3mg DRSP/20µg EE (90% CI) of a) basic characteristics data, b) medical symptoms questionnaire data, c) Piper fatigue scale data, and d) biotransformation and oxidative stress status data

Table S1 Untransformed data of biotransformation efficiency, serum peroxide levels, and antioxidant capacity. *P*arametric statistical analyses was applied. SD, Standard deviation. ES, effect size. Cohen's d value: 0.2, small effect; 0.5, medium effect; 0.8, large effect; 1.3, very large effect. *The BH FDR adjusted p-value was considered significant when <0.1

	**Control**		**COC**		**Control vs COC**	
**Variable**	**Mean**	**(SD)**	**Mean**	**(SD)**	**ES Cohen's d**	**BH FDR Adjusted p-value**
Creatinine *(mmol/l)*	10.47	(6.37)	16.78	(8.99)	0.70	0.036*
Uric acid *(mmol/l)*	1.42	(1.10)	1.63	(1.35)	0.16	0.613
Uric acid:Creatinine ratio	0.18	(0.14)	0.10	(0.06)	0.54	0.066*
Caffeine clearance *(ml/min/kg)*	2.92	(7.84)	0.66	(0.49)	0.29	0.295
APAP-glucuronide *(% recovery)*	27.32	(14.29)	43.24	(13.05)	1.11	0.002*
APAP-sulfate *(% recovery)*	22.40	(9.72)	19.84	(7.39)	0.26	0.378
APAP-mercapturic acid *(% recovery)*	3.68	(2.00)	4.50	(1.61)	0.41	0.247
Salicyluric acid *(% recovery)*	26.36	(11.04)	33.66	(10.17)	0.66	0.066*
Catechol *(mM)*	14.88	(21.02)	9.96	(8.20)	0.23	0.378
2,3-DHBA *(µM)*	3.93	(3.06)	3.16	(2.83)	0.25	0.430
2,5-DHBA *(µM)*	61.77	(44.66)	67.95	(39.28)	0.14	0.641
Carnitine, Total-Free *(mmol/mol creatinine)*	1.63	(0.94)	1.75	(1.40)	0.09	0.722
Acyl-Carnitine, Total *(mmol/mol creatinine)*	2.31	(1.63)	2.95	(1.75)	0.37	0.297
Acyl-Carnitine:Free Carnitine ratio	2.03	(2.33)	3.03	(3.53)	0.28	0.358
PhaseI:PhaseII ratio (Sulfation)	13.24	(26.59)	4.53	(5.24)	0.33	0.247
PhaseI:PhaseII ratio (Glycination)	12.43	(28.06)	2.77	(3.97)	0.34	0.247
PhaseI:PhaseII ratio (Glucuronidation)	15.20	(38.29)	2.39	(3.88)	0.33	0.247
Serum Peroxides *(units)*	72.56	(11.52)	162.89	(24.00)	3.76	<0.001*
FRAP *(units)*	366.21	(58.56)	302.31	(69.92)	0.91	0.008*
GSHt *(µM)*	950.97	(225.3)	877.25	(142.58)	0.33	0.295
